# Longitudinal Assessment of Lipoprotein(a) Levels in Perinatally HIV-Infected Children and Adolescents

**DOI:** 10.3390/v13102067

**Published:** 2021-10-14

**Authors:** Jason G. van Genderen, Malon Van den Hof, Claudia G. de Boer, Hans P. G. Jansen, Sander J. H. van Deventer, Sotirios Tsimikas, Joseph L. Witztum, John J. P. Kastelein, Dasja Pajkrt

**Affiliations:** 1 Department of Pediatric Infectious Diseases, Emma Children’s Hospital, Amsterdam UMC, Location Academic Medical Center, 1100 DD Amsterdam, The Netherlands; m.vandenhof@amsterdamumc.nl (M.V.d.H.); c.g.deboer@amsterdamumc.nl (C.G.d.B.); d.pajkrt@amsterdamumc.nl (D.P.); 2 Department of Experimental Vascular Medicine and Vascular Medicine, Amsterdam UMC, Location Academic Medical Center, 1100 DD Amsterdam, The Netherlands; j.p.jansen@amsterdamumc.nl (H.P.G.J.); j.j.kastelein@amsterdamumc.nl (J.J.P.K.); 3 Department of Gastroenterology, Leiden University Medical Center, 2333 ZD Leiden, The Netherlands; sander.van.deventer@forbion.com; 4 Sulpizio Cardiovascular Center, Division of Cardiovascular Medicine, University California San Diego, La Jolla, CA 92037, USA; stsimikas@health.ucsd.edu; 5 Division of Endocrinology and Metabolism, Department of Medicine, University California San Diego, La Jolla, CA 92093, USA; jwitztum@health.ucsd.edu

**Keywords:** human immunodeficiency virus, cardiovascular disease, apolipoproteins dyslipidemia, atherosclerotic cardiovascular disease

## Abstract

HIV is an independent risk factor of cardiovascular disease (CVD); therefore, perinatally HIV-infected (PHIV) children potentially have a greater CVD risk at older age. Lipoprotein(a) (Lp(a)) is an established risk factor for CVD in the general population. To evaluate a potential increased CVD risk for PHIV children, we determined their lipid profiles including Lp(a). In the first substudy, we assessed the lipid profiles of 36 PHIV children visiting the outpatient clinic in Amsterdam between 2012 and 2020. In the second substudy, we enrolled 21 PHIV adolescents and 23 controls matched for age, sex and ethnic background on two occasions with a mean follow-up time of 4.6 years. We assessed trends of lipid profiles and their determinants, including patient and disease characteristics, using mixed models. In the first substudy, the majority of PHIV children were Black (92%) with a median age of 8.0y (5.7–10.8) at first assessment. Persistent elevated Lp(a) levels were present in 21/36 (58%) children (median: 374 mg/L (209–747); cut off = 300). In the second substudy, the median age of PHIV adolescents was 17.5y (15.5–20.7) and of matched controls 16.4y (15.8–19.5) at the second assessment. We found comparable lipid profiles between groups. In both studies, increases in LDL-cholesterol and total cholesterol were associated with higher Lp(a) levels. A majority of PHIV children and adolescents exhibited elevated Lp(a) levels, probably associated with ethnic background. Nonetheless, these elevated Lp(a) levels may additionally contribute to an increased CVD risk.

## 1. Introduction

Due to effective life-long combination antiretroviral therapy (cART), perinatally HIV-infected (PHIV) children and adolescents are expected to have a near normal life expectancy [1]. However, non-communicable disease manifestations such as metabolic syndrome and cardiovascular disease (CVD) are more likely to occur in people living with HIV compared to the general population [2,3]. In PHIV children and adolescents, cardiovascular complications are also apparent, including structural and functional myocardial dysfunction, increased intima-media thickness and endothelial dysfunction [4]. Although the pathogenesis of HIV-associated CVD risk is incompletely understood, it is hypothesized that multiple pathological mechanisms, including dyslipidemia and insulin resistance [3], direct vascular inflammation [5], as well as certain antiretroviral drugs [6,7], may contribute to this increased CVD risk.

Elevated levels of lipoprotein(a) (Lp(a)) are an established and independent risk factor for CVD in the general population [8]. In HIV-infected adults, longitudinal assessments showed increased Lp(a) levels in association with switching to another antiretroviral treatment, predominantly in participants with pre-existent elevated Lp(a) levels [7,9,10]. Only very limited cross-sectional studies reported on Lp(a) levels in PHIV children [7,11,12,13]. We previously reported higher Lp(a) levels compared to controls matched for ethnic background [11]. Another study found higher Lp(a) levels in PHIV children compared to controls, but these controls were not matched for ethnic background [12]. One small study reported on higher Lp(a) levels in six children after receiving ritonavir [7]. Another study found Lp(a) levels above the reference value in 50% of thirty-seven PHIV children [13].

Lp(a) is mainly genetically determined, with significantly different values between people with a different ethnic background [8,14]. The largest difference is found between people of African and non-African descent [15,16]. In the Netherlands, the majority of PHIV children are born in countries from the sub-Saharan region [17]. To date, there are no data available on longitudinal measurements of Lp(a) in PHIV children and adolescents. A large study found that increased Lp(a) levels are an independent casual risk factor for coronary heart disease [18]. This is important because Lp(a) could be used as a proxy to assess CVD risk in PHIV adolescents since manifestations of CVD usually occur at older age. The identification of (prognostic) factors for increased CVD risk in PHIV children and adolescents may direct the development of novel therapeutic or preventive strategies in order to reduce these risks at older age. To gain insight in the potential prognostic factors for increased CVD risk and possible relation with antiretroviral therapy in PHIV children and adolescents, we determined the lipid profiles—including Lp(a) levels—of PHIV children and adolescents over time and compared them to healthy matched controls.

## 2. Materials and Methods

### 2.1. Study Population

This study consisted of two substudies. In the first substudy, we included PHIV patients who visited the outpatient clinic at the Amsterdam UMC, location Academic Medical Center (AMC), Emma Children’s Hospital in Amsterdam at least on two occasions over a period of eight years between September 2012 and September 2020. For the second substudy, we included participants from a longitudinal cohort study: the Neurological, cOgnitive and VIsual performance in HIV-infected ChildrEn (NOVICE) study. The NOVICE study longitudinally assessed neurological, cognitive and visual performance in PHIV children and adolescents compared to healthy controls that were frequency matched for age, sex, socio-economic status and ethnic background between December 2012 and January 2014 (1st visit) and between February 2017 and July 2018 (2nd visit). The study characteristics were published previously [19].

The study protocols adhered to tenets of the Declaration of Helsinki and followed the guidelines provided by the ethics committee of the Amsterdam University Medical Center, location AMC.

We obtained written informed consent from all parents or legal guardians and all children aged 12 years and older. The NOVICE study was registered with the Dutch Trial Register (Netherlands Trial Register) as NTR4074.

### 2.2. Lipid Profiles

For the first substudy, we retrospectively collected non-fasting lipid profiles in heparin plasma samples obtained during regular medical follow-up at the outpatient clinic. All measurements were immediately performed following the blood draw using the Architect c8000 Abbott (Lake Forest, IL, USA) immunoturbidimetric method. Total cholesterol (TC) levels were measured with the c502 Roche Diagnostics, and high-density lipoprotein cholesterol (HDL-C) and triglycerides (TG) levels with the c702 Roche Diagnostics (Indianapolis, IN, USA). Low-density lipoprotein cholesterol (LDL-C) levels were determined with the Friedewald formula [20].

For the cohort substudy, we collected non-fasting venous blood plasma samples that were stored at –80 °C until further analysis. The following lipid levels were assessed: Lp(a), TC, LDL-C, HDL-C, TG, apolipoprotein B (ApoB), apolipoprotein CIII (ApoCIII) and apolipoprotein E (ApoE) [11].

The reference values were defined as follows: Lp(a) ≥300 mg/L or ≥ 30 mg/dL for the first and second substudy, respectively, TC ≥ 5.2 mmol/L (200 mg/dL), LDL ≥ 3.4 mmol/L (130 mg/dL), HDL ≤ 1 mmol/L (40 mg/dL), TG ≥ 1.2 mmol/L (110 mg/dL) for children aged <10 years and ≥ 1.7 mmol/L (150 mg/dL) for children aged ≥ 10 years, ApoB < 90 mg/dL, ApoCIII ≈ 10 mg/dL and ApoE 3–7 mg/dL [21,22,23]. We previously determined *APOE* genotypes, as they strongly influence Lp(a) levels and additionally account for adequate matching between case and controls [24]. We used the Single Nucleotide Polymorphisms (SNP) method with Light Cycler Roche Diagnostics (Indianapolis, IN, USA) defining the following haplotypes: ε2/ε2, ε2/ε3, ε2/ε4, ε3/ε3, ε3/ε4 and ε4/ε4 [11].

### 2.3. Neuroimaging Details

To explore associations with atherosclerotic macro- and microstructural damage of brain white matter, we used data from our cohort study that we obtained through advanced 3T magnetic resonance image (MRI) scans (Ingenia, Philips Healthcare, Best, The Netherlands). White matter hyperintensities (WMH) were previously determined by processing fluid attenuated inversion recovery (FLAIR) scans by means of a semi-automatic segmentation technique. One investigator (MvdH) manually segmented MRI under supervision of an experienced neuroradiologist using ITK-SNAP version 3.4.0 (Philadelphia, PA, USA and Salt Lake City, UT, USA): a segmentation software tool [25,26]. We also investigated associations with the following MRI parameters: Fractional Anisotropy (FA), Mean Diffusivity (MD), Axial Diffusivity (AD) and Radial Diffusivity (RD). These parameters were derived by processing diffusion tensor imaging (DTI) MRI scans and can indicate brain white matter microstructure damage.

### 2.4. HIV Characteristics

Historical cART- and HIV-related characteristics of the PHIV participants were collected from patient records or provided by the Dutch HIV monitoring foundation [27]. We defined cART as the use of at least three antiretroviral drugs from a minimum of two drug classes. In all controls, an HIV negative status was re-confirmed as reported previously [19].

### 2.5. Statistical Analyses

We performed statistical analyses using R version 3.5.1 (R Core team, Vienna, Austria) [28]. We considered *p* < 0.05 as statistically significant. We compared demographic variables between PHIV children and controls using the Mann–Whitney *U* test for continuous data and Fisher’s exact test for categorical data.

We used mixed models to determine lipid levels over time and to assess associations of Lp(a) levels with the following demographic, HIV- and cART-related characteristics: age, sex, body mass index (BMI), other lipids, *APOE* genotypes, different antiretroviral treatment regimens and MRI parameters: WMH, FA, MD, AD and RD. We calculated the intra-individual variance of Lp(a) using mixed models (R package: *nlme*) with which we created a multilevel model with heterogeneous variance [29].

## 3. Results

### 3.1. Participants’ Characteristics

#### 3.1.1. Retrospective Longitudinal Substudy

Table 1 shows the participants’ characteristics at the time of their first and last lipid profile assessment. We included 36 PHIV children and adolescents with a median age of 8.0 years (IQR: 5.7–10.8). Of the included patients, 24 (67%) were boys and 33 (92%) had a Black ethnic background. All patients received cART, with either an NNRTI (50%) or a PI-based (47%) regimen at the first assessment.

#### 3.1.2. Cohort Substudy

Table 2 shows the participants’ characteristics of PHIV adolescents and matched controls at the time of the second enrollment, and also some characteristics at the time of the first enrollment for comparison purposes. The number of participants who provided consent for follow-up was similar between groups: 21/34 (62%) PHIV and 23/37 (62%) controls, respectively. For the 13 PHIV and 14 controls, reasons to not participate were: unwillingness to participate (nine in both groups), inability to contact (five controls) or relocation (two PHIV). Three out of 21 PHIV adolescents were also part of the first longitudinal study. At baseline, there were no differences in Lp(a) levels and *APOE* genotypes between PHIV adolescents and controls who participated at follow-up and those who dropped out.

The median age (in years) at the second enrollment was similar between groups: 17.5 (IQR: 15.5–20.7) and 16.4 (IQR: 15.8–19.5) in PHIV adolescents and controls, respectively. There were no significant differences in participants’ characteristics. Of the participating PHIV adolescents, 19 (90%) had an undetectable HIV viral load (VL) at the time of the second assessment (at the end of the follow-up period), and two PHIV adolescents had an HIV VL of 1515 and 32,996 copies/mL. Fifteen (71%) PHIV adolescents had a consistent undetectable HIV VL during the entire follow-up period.

### 3.2. Lipid Profiles over Time

#### 3.2.1. Retrospective Longitudinal Substudy

**Figure 1:**
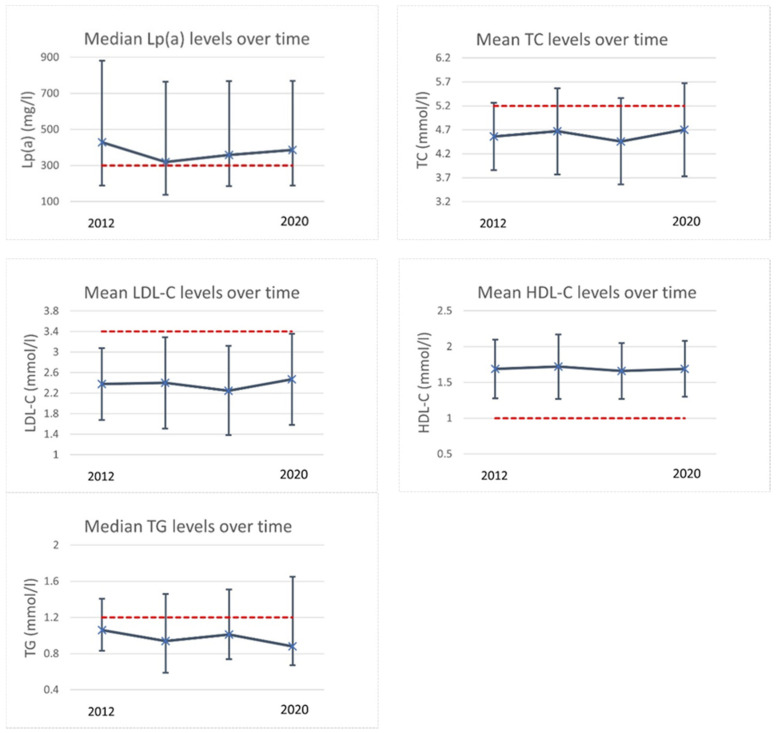
Lipid trends in retrospective longitudinal substudy. This figure shows the lipid trends of all 36 participants in the retrospective longitudinal substudy. Values are either median with interquartile range or mean with +/- one standard deviation. Red dashed lines show the reference values. Reference values were: Lp(a) > 300 mg/L, TC ≥ 5.2 mmol/L (200 mg/dL), LDL ≥ 3.4 mmol/L (130 mg/dL), HDL ≤ 1 mmol/L (40 mg/dL), TG ≥ 1.2 mmol (110 mg/dL) for children aged < 10 years and ≥ 1.7 mmol (150 mg/dL) for children aged ≥ 10 years. Abbreviations: HDL-c = high-density lipoprotein cholesterol; LDL-C = low-density lipoprotein; Lp(a) = lipoprotein(a); TC = total cholesterol; TG = triglycerides.

Figure 1 shows the trends of lipid profiles of 36 PHIV children of the retrospective longitudinal substudy. Over time, the median Lp(a) level was 374 mg/L (IQR: 209–747). Of these 36 PHIV patients, 21 (58%) had an Lp(a) value above the reference value during the entire period with a median Lp(a) level of 733 mg/L (IQR: 460–995). The intra-individual variance of Lp(a) measurements was 29%.

#### 3.2.2. Cohort Substudy

**Figure 2:**
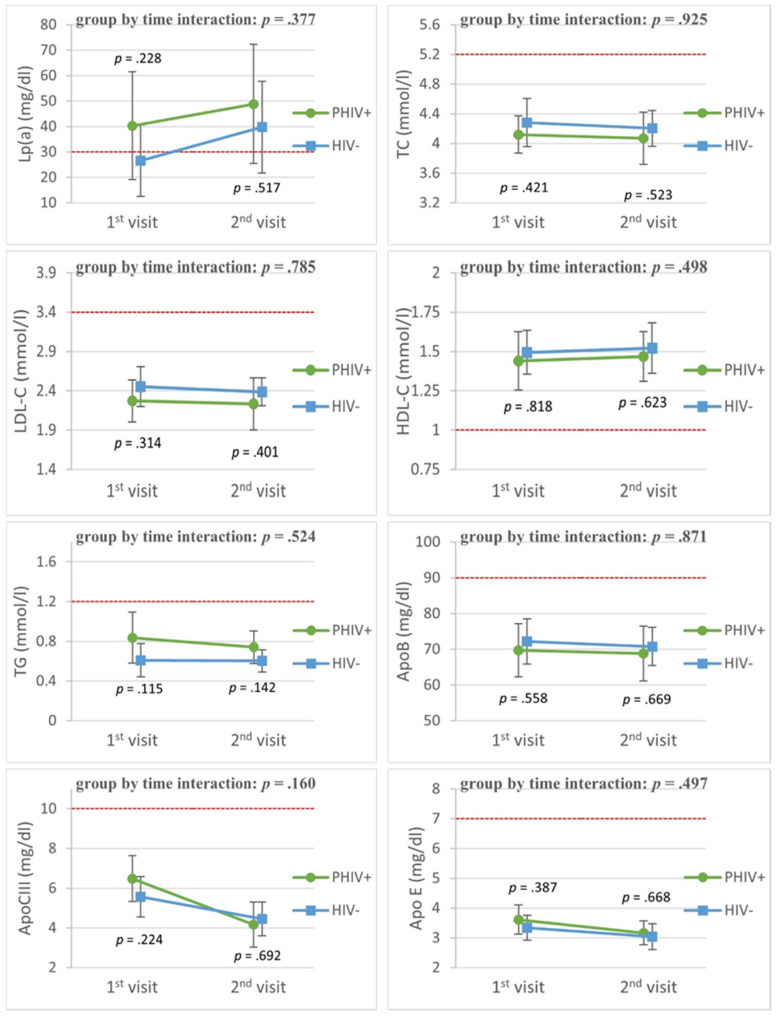
Lipid trends in cohort substudy. The graphs show longitudinal changes in lipid profiles of PHIV+ adolescents compared to controls matched for age, sex, ethnic background and socio-economic status. The figures show plotted least square means and their 95% confidence interval. P values are the unadjusted group by time interaction and cross-sectional difference at first and second visit. The red dashed lines show the reference values. The figures show the following lipids (with reference value in brackets): Lp(a) = lipoprotein(a) (analysis performed with logarithmical values); ApoB = apolipoprotein B; ApoCIII = apolipoprotein CIII; ApoE = apolipoprotein E; TC = total cholesterol; LDL-C = low-density lipoprotein cholesterol; HDL-C = high-density lipoprotein cholesterol; TG = triglycerides. Reference values: Lp(a) = 30 mg/dL, TC ≥ 5.2 mmol/L (200 mg/dL), LDL ≥ 3.4 mmol/L (130 mg/dL), HDL ≤ 1 mmol/L (40 mg/dL), TG ≥ 1.2 mmol (110 mg/dL) for children aged < 10 years and ≥ 1.7 mmol (150 mg/dL) for children aged ≥ 10 years, ApoB < 90 mg/dL, ApoCIII ≈ 10 mg/dL, ApoE 3–7 mg/dL.

Figure 2 shows the lipid profiles of PHIV adolescents and matched controls in the cohort study. The mean Lp(a) levels were higher in PHIV adolescents, although there was no significant difference with controls. Over time, there were similar levels of ApoB, ApoCIII, ApoE, TC, LDL-C, HDL-C and TG. ApoCIII showed a significant decline over time in both groups (time coefficient: –1.18, *p* = 0.02).

### 3.3. Factors Associated with Lp(a)

#### 3.3.1. Retrospective Longitudinal Substudy

In the first substudy, we found that in PHIV children higher levels of TC and LDL-C were individually associated with higher Lp(a) levels, after adjusting for BMI (Table 3). The *APOE* haplotype ε2/ε2 was univariably associated with lower Lp(a) levels. We found no significant associations when assessing all significant variables in a multivariable analysis (Table S1).

#### 3.3.2. Cohort Substudy

In the cohort substudy, TC and LDL-C were positively associated with Lp(a) levels in both PHIV adolescents and controls (Table 4). We found that the ε2/ε4 haplotype was associated with higher Lp(a) levels. BMI was positively associated with Lp(a) levels in PHIV adolescents. We found no associations between brain MRI parameters, HIV-associated variables and Lp(a).

## 4. Discussion

In this study, we found persistently elevated Lp(a) levels in 21 (58%) PHIV children over a period of eight years. Over time, higher levels of LDL-C and TC were associated with higher Lp(a) levels, after adjusting for BMI. In the longitudinal cohort study, we found no significant difference in lipid levels between PHIV adolescents and matched controls over time, with a mean follow-up time of 4.6 years. Over time, higher TC and LDL-C levels were associated with higher Lp(a) levels in both cases and controls. In PHIV adolescents, a higher BMI was associated with higher Lp(a) levels.

Here, we report elevated Lp(a) levels similar to previous studies in PHIV children and adolescents [11,12,13]. We did not find a significant difference in Lp(a) over time between PHIV adolescents and matched controls. This finding suggests that HIV infection is not associated with higher Lp(a) levels in our cohort. However, this lack of association may be explained by our small sample size with subsequent large confidence intervals and comparable ethnic backgrounds. Moreover, as we showed that Lp(a) levels can fluctuate over time—statistically determined with the intra-individual variance—the small number of lipid profile assessments may have hampered the detection of an association between Lp(a) levels and HIV infection. In the control group, we found a median Lp(a) level at follow-up above the reference level. This finding could be explained by their ethnic background [8]. A firm conclusion in regard to their CVD risk cannot be drawn with just two Lp(a) measurements.

HIV infection is an independent risk factor for CVD; elevated Lp(a) levels as found in the majority of PHIV patients in our first substudy may therefore add to their CVD risk [2]. Lp(a) levels differ between people with different ethnic backgrounds, with the highest levels found in Black individuals [8,14]. For Black individuals, Lp(a) levels above 30 mg/dL are indicative of increased risk for coronary heart disease [30]. A very large recent study also found higher Lp(a) levels to be associated with atherosclerotic CVD, irrespective of ethnic background [16]. Another study reported contrasting results in African participants; however, that study had lower power (17%) to detect this association [31]. Indeed, older reports demonstrate associations between increased Lp(a) levels during childhood and a family history of premature heart attack [15,32].

It is generally assumed that Lp(a) levels in the general population are stable [14], although fluctuating levels in both children and adolescents with familial hypercholesterolemia or parental myocardial infarction have been reported [15,33]. A study in adults reported mildly fluctuating Lp(a) levels [34]. Unexpectedly, we detected highly fluctuating Lp(a) levels in PHIV children and adolescents, and these data suggest that a single Lp(a) level measurement in PHIV patients may not be sufficient as a potential prognostic risk factor for CVD in this population.

Increased Lp(a) levels were previously shown after the initiation of antiretroviral treatment [9], and we found that the use of NNRTI was univariably associated with higher Lp(a) levels. This association was not detected after adjusting for BMI, potentially indicating confounding. It is possible that cART underlies both weight gain and an increase in Lp(a) levels [35].

It remains pivotal to investigate cART regimens in relation to CVD risk as protease inhibitors have been shown to increase CVD risk [6,7]. Our findings suggest that Lp(a) levels might be considered as one of the factors when optimizing cART regimens, but in order to definitely link cART-related LP(a) changes to a potential increased CVD risk in PHIV children and adolescents, larger outcome studies would be required.

We found a significant association of Lp(a) levels with certain *APOE* haplotypes, which is in concordance with the results of a large study indicating that *APOE* genotypes influence Lp(a) levels [24]. We performed a large multivariable association, including *APOE* genotypes; however, these results should be interpreted with caution due to the low number of participants and presumed overfitting of the model [36].

In the cohort study, we found that higher LDL-C and TC levels were associated with higher Lp(a) levels in both cases and controls, and these results could indicate that Lp(a) cholesterol contributes to a higher detection of LDL-C [37]. Although there are calculations for the Lp(a)-cholesterol contribution to LDL-C, a recent study showed that these calculations are not likely to be accurate [38]. In PHIV adolescents, a higher BMI was associated with higher Lp(a) levels, as was demonstrated in a large study in obese children [39].

We assessed the association between WMH and Lp(a) due to the atherosclerotic origin of WMH [40]. We found no association between WMH and Lp(a). The low absolute volume of WMH in our participants could have hampered the detection of this association [26]. We also found no association between white matter microstructure and Lp(a). We assessed this association as previous studies reported mixed results and suggested that PHIV children possibly have a higher prevalence of white matter microstructure damage compared to matched controls [26,41,42,43].

### Strengths and Limitations

Our study is the first to report on longitudinal Lp(a) levels in PHIV children and adolescents leading to an increased understanding of their CVD risk. We analyzed a great number of lipid profile measurements over a long period of time. To explore possible associations, we matched the PHIV patient group to a well-defined ethnic control group.

Our study has some important limitations. First, the retrospective longitudinal substudy did not contain a control group, precluding multiple lipid profile comparisons between PHIV children and controls over time. Both substudies have small sample sizes due to the low prevalence of PHIV pediatric patients in the Netherlands [27], reducing the power to generalize our results or to perform additional multivariable analyses. We analyzed non-fasting plasma samples, which potentially limits the interpretation of all lipid profiles, although Lp(a) is shown not to be influenced by food intake [44]. In the cohort study, the samples collected at baseline were stored at –80 °C for a longer period than the samples collected at follow-up and could have led to lower Lp(a) levels at baseline. Our study did not include other surrogate markers for CVD risk, such as oxidized phospholipids [45] or carotid intima thickness. Although higher Lp(a) levels are associated with endothelial dysfunction, the association between Lp(a) and carotid intima thickness is not established [46,47]. We also did not measure the LPA gene kringle repeat number, which strongly affects Lp(a) levels, but our small sample size would render such analysis rather meaningless.

## 5. Conclusions

More than half of PHIV patients visiting our outpatient clinic have elevated Lp(a) levels and thus may be at increased risk of developing CVD at an older age, as both elevated Lp(a) levels and HIV diagnosis are independently associated with increased CVD risk [2,8]. The ethnic background of our participants possibly contributed to higher Lp(a) levels. Currently, effective Lp(a) lowering therapies are not available, and the association between HIV and increased Lp(a) remains to be confirmed. Nonetheless, the current findings underscore the importance of a careful follow-up of PHIV patients in order to detect early manifestations of CVD and early counseling to reduce behavioral risk factors for CVD. To evaluate whether PHIV patients have a different CVD risk profiles than behaviorally acquired HIV-infected patients, comparison studies are needed. Ultimately, innovative Lp(a) lowering therapeutic strategies, which are currently being developed, may become available for the treatment of PHIV patients at risk [48].

## Figures and Tables

**Table 1:** Baseline participants’ characteristics of retrospective longitudinal substudy.

	First Assessment (n = 36)	Last Assessment (n = 36)
Age (y)	8.0 (5.7–10.8)	11.1 (3.6)
Male sex	24 (67%)	
Ethnic background		
Black	33 (92%)	
Other	3 (8%)	
Adopted	30 (83%)	
Height (m)	1.12 (0.95–1.30)	1.47 (1.33–1.62)
Weight (kg)	19.8 (14.4–30.2)	42.0 (29.2–52.5)
Body Mass Index	16.2 (15.2–17.8)	18.7 (16.2–21.5)
*APOE* genotypes		
ε2/ε2	1 (5%)	
ε2/ε3	4 (17%)	
ε2/ε4	2 (8%)	
ε3/ε3	10 (42%)	
ε3/ε4	4 (17%)	
ε4/ε4	3 (13%)	
Age at start cART (y)	2.8 (0.3–5.8)	
CDC category		
NA	19 (83%)	
B	1 (4.3%)	
C	3 (13%)	
HIV viral load (<40 copies/mL)		
Undetectable	32 (89%)	36 (100%)
Detectable	4 (11%)	0 (0%)
* CD4+ T cell nadir (cells/µL)	0.82 (SD: 0.31)	
CD4+ T cell nadir *Z* score	0.03 (SD: 1.1)	
CD4+ T cell nadir %	35% (31–39%)	
* HIV zenith (log copies/mL)	3.6 (2.5–5.4)	
cART regiment, no(%)		
Backbone + NtRTI	1 (3%)	0 (0%)
Backbone + NNRTI	18 (50%)	1 (3%)
Backbone + PI	17 (47%)	10 (28%)
Backbone + INSTI	0 (0%)	25 (69%)
* Exposure to cART		
PI ever used		23 (64%)
Duration (y)		3.41 (2.35–4.59)
INSTI ever used		24 (67%)
Duration (y)		1.78 (1.34–2.42)
NNRTI ever used		20 (56%)
Duration (y)		2.68 (1.32–3.95)

Values are noted in number and percentage, mean and standard deviation or median and interquartile range. * indicates values extracted for duration of follow-up. CD4+ T cell Z score is age-adjusted. Abbreviations: CDC = Center for Disease Control and Prevention; NA = no or minimal symptoms of AIDS; B = moderate symptoms; C = severe symptoms or AIDS; HIV = human immunodeficiency virus; INSTI = Integrase Strand Transfer Inhibitor; IQR = inter-quartile range; kg = kilogram; m = meter; NNRTI = Non-Nucleoside Reverse-Transcriptase Inhibitor; NtRTI = Nucleotide Reverse-Transcriptase Inhibitor; PI = Protease Inhibitor; SD = standard deviation; y = years. Deviation of number: HIV zenith (19); CDC (23); height (33 and 30); weight (33 and 31); BMI (30 and 33); APOE (24).

**Table 2:** Participants’ characteristics of cohort substudy.

	PHIV	CONTROLS	
			*p*
Follow-up rate	62%	62%	
Follow-up time	4.65 (0.33)	4.55 (0.33)	0.343 ^X^
Age (y)			
At first enrollment	13.4 (10.9–15.6)	12.1 (11.1–15.2)	0.655 ^Z^
At second enrollment	17.5 (15.5–20.7)	16.4 (15.8–19.5)	0.526 ^Z^
Male sex	12 (57%)	9 (39%)	0.365 ^Y^
Ethnic background			0.606 ^Y^
Black	17 (81%)	18 (78%)	
White	0 (0%)	2 (9%)	
Other	4 (19%)	3 (13%)	
Height (m)	1.66 (0.13)	1.69 (0.09)	0.297 ^X^
Weight (kg)	56.3 (49.0–69.5)	63.0 (56.0–67.4)	0.109 ^Z^
Body Mass Index	20.4 (19.2–22.3)	22.2 (19.9–26.3)	0.137 ^Z^
Overweight or obese *			
At first enrollment	1 (5%)	1 (4%)	0.999 ^Y^
At second enrollment	2 (10%)	7 (30%)	0.137 ^Y^
Blood Pressure (mmHg)			
Systolic	114 (109–125)	119 (114–124)	0.484 ^Z^
Diastolic	65 (56–73)	64 (59–74)	0.843 ^Z^
Lifestyle			
Ever Smoked	7 (33%)	5 (22%)	0.504 ^Y^
*APOE* genotypes			0.466 ^Y^
ε2/ε2	0 (0%)	0 (0%)	
ε2/ε3	3 (17%)	2 (10%)	
ε2/ε4	0 (0%)	3 (14%)	
ε3/ε3	9 (50%)	10 (48%)	
ε3/ε4	6 (33%)	5 (24%)	
ε4/ε4	0 (0%)	1 (5%)	
Age at HIV diagnosis (y)	1.72 (0.83–4.16)		
CDC			
NA	8 (38%)		
B	8 (38%)		
C	5 (24%)		
Age at treatment initiation (y)	2.52 (1.20–5.97)		
cART duration (y)	14.86 (9.51–19.57)		
cART use			
At 1st enrollment	20 (95%)		
At 2nd enrollment	20 (95%)		
Current regimen			
Backbone + INSTI	12 (60%)		
Backbone + PI	4 (20%)		
Backbone + NNRTI	4 (20%)		
CD4^+^ nadir	460 (300–570)		
CD4^+^ *Z* score	−0.82 (0.61)		
HIV zenith (log copies/mL)	5.5 (4.9–5.8)		
Undetectable HIV viral load			
At 1st enrollment	20 (95%)		
At 2nd enrollment	19 (90%)		
During entire follow-up	15 (71%)		

Values are noted in number and percentage, mean and standard deviation or median and inter-quartile range. CD4+ nadir Z score is age-adjusted. * Based on BMI for age by CDC. Statistical tests: X = Student’s *t* test; Y = Fisher’s exact test; Z = Mann–Whitney U test. Abbreviations: CDC = Center for Disease Control and Prevention; NA = no or minimal symptoms of AIDS; B = moderate symptoms; C = severe symptoms or AIDS; HIV = human immunodeficiency virus; INSTI = Integrase Strand Transfer Inhibitor; IQR = inter-quartile range; kg = kilogram; m = meter; n = amount; NNRTI = Non-Nucleoside Reverse-Transcriptase Inhibitor; NtRTI = Nucleotide Reverse-Transcriptase Inhibitor; PI = Protease Inhibitor; SD = standard deviation; y = years. Deviation in number (PHIV): height (19); weight (19); BMI (19) systolic (19); diastolic (19); APOE (18); treatment initiation and duration (18); CD4+ (19); HIV zenith (18). Deviation in number controls: systolic (18); diastolic (18); APOE (21).

**Table 3:** Factors associated with Lp(a) in retrospective longitudinal substudy.

	Lp(a) of PHIV+ Visiting Outpatient Clinic					
	Univariable Analyses			Analyses (Adjusted for BMI)		
	Coefficient	95%CI	*p*	Coefficient	95%CI	*p*
Age	0.99	0.98–1.01	0.402			
Male sex	0.53	0.26–1.01	0.079			
BMI (kg/m^2^)	0.98	0.96–1.02	**0.029**	-	-	-
PI use	0.97	0.89–1.05	0.401			
INSTI use	0.96	0.90–1.02	0.171			
NNRTI use	1.10	1.03–1.19	**0.007**	1.07	0.99–1.16	0.111
Lipids (mmol/L)						
TC	1.12	1.06–1.18	<**0.001**	1.14	1.08–1.20	<**0.001**
LDL-C	1.11	1.05–1.18	<**0.001**	1.14	1.07–1.21	<**0.001**
HDL-C	1.03	0.92–1.16	0.570			
TG	0.99	0.97–1.05	0.698			
*APOE* genotypes						
ε2/ε2	0.18	0.03–0.98	**0.049**			
ε2/ε3	1.17	0.45–3.03	0.732			
ε2/ε4	2.12	0.61–7.39	0.229			
ε3/ε3 *	-	-	-			
ε3/ε4	0.79	0.30–2.05	0.623			
ε4/ε4	1.28	0.41–3.71	0.636			

Linear mixed models with both univariable and multivariable analyses showing associations over time. Bold numbers show significant *p* values. * this variable is used as reference. The three multivariate analyses show individual associations for NNRTI use, increase in TC and LDL-C. Each model is adjusted for BMI. Abbreviations: HDL-C = high-density lipoprotein cholesterol; INSTI = Integrase Strand Transfer Inhibitor; LDL-C = low-density lipoprotein cholesterol; NNRTI = Non-Nucleoside Reverse-Transcriptase Inhibitor; PI = Protease Inhibitor; TC = total cholesterol; TG = triglycerides.

**Table 4:** Factors associated with Lp(a) in cohort substudy.

	Lp(a) of PHIV Adolescents AND Controls					
	Univariable Analyses with Mixed Models			Multivariable Analyses with Mixed Models		
	Coefficient	95%CI	*p*	Coefficient	95%CI	*p*
HIV diagnosis	2.41	0.85–6.75	0.106			
Age	1.03	0.96–1.10	0.397			
Male Sex	1.00	0.42–2.38	0.999			
Systolic BP (mmHg)	1.00	0.99–1.02	0.744			
BMI (kg/m^2^)	1.03	0.95–1.11	0.512			
Lipids (mmol/L)						
**TC**	1.6	1.00–2.59	**0.048**	0.88	(0.31–2.74)	0.832
HDL-C	1.02	0.42–1.10	0.958			
**LDL-C**	2.29	1.28–3.90	**0.004**	2.35	(0.53–7.78)	0.231
TG	0.65	0.38–1.08	0.101			
*APOE* genotypes						
ε2/ε2	-	-	-			
ε2/ε3	0.54	0.14–2.16	0.423			
ε2/ε4	0.04	0.01–0.17	**0.001**	0.04	(0.007–0.24)	**0.004**
ε3/ε3 *	-	-	-			
ε3/ε4	0.54	0.20–1.52	0.285			
ε4/ε4	0.68	0.06–8.25	0.779			
MRI parameters						
WMH volume (mm^3^)	1.00	0.90–1.00	0.123			
FA	0.01	−0.02 to 0.03	0.527			
MD	−0.02	−0.05 to 0.01	0.128			
AD	−0.03	−0.06 to 0.01	0.168			
RD	−0.03	−0.06 to 0.01	0.141			
**Lp(a) of PHIV+ adolescents**						
Univariable analyses with mixed models						
	coefficient	95%CI	*p*			
HIV VL zenith (log) c/mL	1.66	0.25–11.2	0.601			
CD4^+^ nadir Z-score	1.22	0.55–2.72	0.624			
Duration of cART (years)	1.01	0.92–1.11	0.836			
CDC category						
B	1.40	0.51–3.86	0.535			
C	1.94	0.61–6.17	0.290			
Undetectable VL at both measurements	0.79	0.29–2.16	0.652			
**BMI (kg/m^2^)**	1.07	1.03–1.12	**0.002**			
PI use	0.90	0.53–1.46	0.675			
INSTI use	1.11	0.89–1.38	0.381			
NNRTI use	0.90	0.72–1.16	0.434			

Linear mixed models with univariable analyses showing associations with Lp(a) over time. Bold numbers show significant *p* values. * this variable is used as reference. We also performed multivariable analyses with significant variables in PHIV adolescents and controls. DTI parameters were adjusted with a fixed factor (1000) to avoid very small coefficients. Abbreviations: AD = Axial Diffusivity; cART = combination antiretroviral therapy; BMI = body mass index; BP = blood pressure; CDC = Center for Disease Control and Prevention (B = moderate symptoms; C = severe symptoms or AIDS); FA = fractional anisotropy; HDL-C = high-density lipoprotein cholesterol; HIV = human immunodeficiency virus; LDL-c = low-density lipoprotein cholesterol; INSTI = Integrase Strand Transfer Inhibitor; log = logarithmic value; MD = Mean Diffusivity; NNRTI = Non-Nucleoside Reverse-Transcriptase Inhibitor; PI = Protease Inhibitor; RD = Radial Diffusivity; TC = total cholesterol; TG = triglycerides; VL = viral load; WMH = white matter hyperintensities.

## Data Availability

Data cannot be shared publicly due to (potentially) sensitive patient information. Data are available upon request from either the Medical Ethics Committee of Amsterdam UMC or the corresponding author.

## Supplementary Materials

The following are available online at https://www.mdpi.com/article/10.3390/v13102067/s1, Table S1: A. Multivariable analyses in PHIV children and adolescents from first substudy.
