# Supplementary material for: Longitudinal Assessment of Lipoprotein(a) Levels in Perinatally HIV-Infected Children and Adolescents
Source: Viruses. 2021 Oct 14;13(10):2067. doi: 10.3390/v13102067 (PMC8539147; doi:10.3390/v13102067)
Supplement: Supplementary file 1 [file viruses-13-02067-s001.zip › viruses-1386804-supplementary.pdf]

viruses

Supplementary Materials

Table S1. A. Multivariable analyses in PHIV children and adolescents from first substudy.

	Coefficient	(95%CI)	p	Coefficient	(95%CI)	*p*
TC (mmol/l)	1.09	(0.99-1.20)	.077	1.06	(0.93-1.20)	.381
NNRTI use	1.06	(0.98-1.15)	.171	1.03	(0.93-1.14)	.579
BMI (kg/m^2)	0.98	(0.95-1.00)	.108	0.99	(0.96-1.01)	.277
LDL-C (mmol/l)	1.05	(0.95-1.17)	.348	1.09	(0.95-1.23)	.230
ε2/ε2 haplotype				0.18	(0.04-0.91)	.079

Multivariable analyses using mixed models with all significant variables. Results might be biased due to overfitting. Abbreviations: BMI = body mass index; CI = confidence interval; TC = total cholesterol; LDL-C = low-density lipoprotein cholesterol; NNRTI = Non-Nucleoside Reverse-Transcriptase Inhibitor.
